# Toward a policy ecology of implementation of evidence-based practices in public mental health settings

**DOI:** 10.1186/1748-5908-3-26

**Published:** 2008-05-16

**Authors:** Ramesh Raghavan, Charlotte Lyn Bright, Amy L Shadoin

**Affiliations:** 1 George Warren Brown School of Social Work, and Department of Psychiatry, School of Medicine, Washington University in St. Louis, St. Louis, MO, USA; 2 George Warren Brown School of Social Work, Washington University in St. Louis, St. Louis, MO, USA; 3 Social Metrics, Inc., Huntsville, AL, USA

## Abstract

**Background:**

Mental health policymaking to support the implementation of evidence-based practices (EBPs) largely has been directed toward clinicians. However, implementation is known to be dependent upon a broader ecology of service delivery. Hence, focusing exclusively on individual clinicians as targets of implementation is unlikely to result in sustainable and widespread implementation of EBPs.

**Discussion:**

Policymaking that is informed by the implementation literature requires that policymakers deploy strategies across multiple levels of the ecology of implementation. At the organizational level, policies are needed to resource the added marginal costs of EBPs, and to assist organizational learning by re-engineering continuing education units. At the payor and regulatory levels, policies are needed to creatively utilize contractual mechanisms, develop disease management programs and similar comprehensive care management approaches, carefully utilize provider and organizational profiling, and develop outcomes assessment. At the political level, legislation is required to promote mental health parity, reduce discrimination, and support loan forgiveness programs. Regulations are also needed to enhance consumer and family engagement in an EBP agenda. And at the social level, approaches to combat stigma are needed to ensure that individuals with mental health need access services.

**Summary:**

The implementation literature suggests that a single policy decision, such as mandating a specific EBP, is unlikely to result in sustainable implementation. Policymaking that addresses in an integrated way the ecology of implementation at the levels of provider organizations, governmental regulatory agencies, and their surrounding political and societal milieu is required to successfully and sustainably implement EBPs over the long term.

## Background

Mental health policymaking in the past several decades has explicitly encouraged the adoption and implementation of specific evidence-based practices (EBPs). Purchasers (such as Medicaid agencies) and regulators (such as departments of mental health) have established lists of preferred therapies, have conducted provider profiling, and have provided training and technical assistance – all in an attempt to ensure that best available interventions are being delivered by clinicians to their clients. State agencies, therefore, largely seem to be taking a *clinical *approach to the implementation of EBPs. Conversely, the emerging literature within implementation science suggests that implementation requires a systemic, or *ecological*, approach [1]. By ignoring this ecology, current policymaking to support the implementation of EBPs is itself not evidence-based.

**Figure 1:**
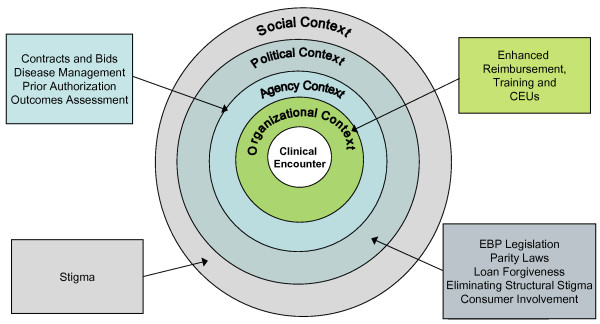
A Policy Ecology of Implementation.

In this article – directed toward policymakers and implementation researchers in public mental health settings – we argue that mandating the use of EBPs by individual clinicians and provider organizations, or narrowly focusing on effecting change within individual organizations, is unlikely to result in their successful and sustainable implementation unless the broader ecology within which these interventions are delivered is also supported. Following a brief overview of this implementation ecology, we present a framework to operationalize this ecology and illustrate it in Figure 1. We end by highlighting potential strategies at each level of this framework that policymakers can deploy in order to support implementation of EBPs and summarize these strategies in Table 1.

**Table 1:** Summary of Strategies for Policymakers

*Level in the Policy Ecology*	*Strategy*
Provider organization	Developing flexible and enhanced reimbursement strategies that accommodate the increased costs of EBP implementation.
	Re-engineering continuing education units to support training in EBPs, auditing and feedback, and disallowing of certain courses for CEU credit.
Regulatory or purchaser agency	Influencing the type of care purchased by changing contracting and bidding procedures.
	Considering expansion of disease management programs as a model for comprehensive EBP implementation.
	Using procedural mechanisms such as prior authorization to support specific EBPs.
	Developing and measuring client-level outcomes to assess the effectiveness of EBPs, and aligning purchasing to the attainment of these outcomes.
Political	Carefully considering enabling legislation to purchase EBPs.
	Legislating mental health parity, and supporting the reduction of stigma and discrimination of individuals with mental health diagnoses.
	Legislating loan forgiveness programs for providers who adopt and promote the use of EBPs.
	Identifying and eliminating structural stigma in all legislation.
	Involving consumer advocates at all levels of implementation.
Social	Reducing stigma and discrimination that can prevent access to needed mental health services, including EBPs.

### The march toward evidence

Quality improvement within mental health services has long been a goal of policy. Operationalization of quality improvement efforts has occurred largely at the level of individual clinicians and their clients through the development and deployment of specific interventions backed by research evidence, clinical judgment, and client preferences. Pioneered at McMaster University as 'evidence-based medicine' [2,3], applications of this approach to mental health have resulted in various EBPs [4,5]. These practices are often packaged with manuals and other materials suitable for demonstrating a particular practice to clinicians [6-9].

Policymaking directed toward mental health quality improvement evolved to support these clinical efforts. Government agencies have supported the development and use of clinical guidelines to standardize care [10,11]. They also have released reports on various aspects of quality [12] and have supported the widespread dissemination and use of evidence-based mental health interventions [7]. States have also incentivized (*i.e*., created a reward structure for using) EBPs – Oregon's passage of SB 267 in 2003 requiring the state to spend an increasing share of its budget in purchasing specified interventions is one example [13]. States also have required the use of particular clinical protocols (*e.g*. Texas' emphasis on the use of medication algorithms) [14]. The District of Columbia's Department of Mental Health has adopted a policy to support 'evidence-based psychotherapy,' which requires that all psychotherapy provided to clients in the District appear on a list maintained by the department [15]. Today, the number of individuals receiving interventions that are evidence-based is one of the Substance Abuse and Mental Health Services Administration's (SAMHSA) National Outcome Measures [16]. Efforts by governmental agencies to improve quality in Britain [17], Brazil [18], and Germany [19], to name a few examples, have also been largely focused at the clinical level.

## Discussion

### The ecology of implementing EBPs

In FY 2005, SAMHSA began funding mental health transformation state incentive grants [20,21]. In terms of policy, these grants marked a shift in thinking from incentivizing the development and deployment of specific interventions, to incentivizing the infrastructure necessary for appropriate service delivery. This shift in policy was to some degree influenced by the wealth of literature that has accumulated in recent years on institutional frameworks [22,23] and organizational factors that can guide implementation efforts [24-26]. This literature suggests that, although the clinical encounter is where EBPs are delivered, efforts to promote the implementation of such practices should focus on the wider context of service delivery [24,27,28]. Collectively, this literature articulates an ecology of mental health intervention ranging from the clinical encounter to the social context of mental health service delivery. As articulated by Vijay Ganju [29]:

This perspective goes beyond the adoption of the EBP by an individual practitioner or organization and includes the notion of broader, systemwide availability of EBPs and their integration into existing systems of care. ... The model that emerges related to the ultimate, broad based adoption depends on the nature of the EBP ..., the consumer ..., the practitioner ..., the organizational matrix within which the practitioner operates ... and the public mental health authority, or purchaser .... The implication is that each of these levels must be adequately addressed for sustained, systemwide uptake of an EBP.

Implementation spans the set of activities necessary to successfully and sustainably apply with high fidelity an intervention of known efficacy within community-based clinical settings. These activities are contextual, involving the organization within which services are delivered, the regulatory and funding environment operant upon the organization, a political milieu that supports mental health service delivery, and societal norms and subcultures that affect consumers' access to EBPs. Therefore, policymaking that is focused exclusively toward clinicians is unlikely to be sustainable; instead, policymakers need to align the effects of policy action across all of these contexts in order to produce 'sustained, systemwide uptake' of EBPs. Implementation researchers designing and conducting implementation studies even at the level of a single organization need to be cognizant of influences at multiple levels of the organization that can affect their chances of success. In the remainder of this article, we describe a policy ecology framework for EBP implementation, and identify policy levers (*i.e*. strategies that policymakers can deploy) at each of these contexts – other than at the level of the individual client/practitioner encounter – that, when addressed, can result in sustainable uptake of EBPs (Figure 1).

### Policy levers at the organizational level

Organizations – ranging from small mental health practice associations to large, multidisciplinary mental health facilities – form the immediate context within which most clinicians deliver mental health interventions to consumers. Attempting to deliver new evidence-based interventions within organizations is associated with several challenges [7,30]; however, once organizations determine that a particular function, such as delivering EBPs to a particular population, is part of their core mission, they tend to protect these technical functions from what Robert Rosenheck describes as the 'stormy sea of organizational process' [30]. In addition to protecting core functions, some 'learning organizations' [31] actively seek out ways to improve their core missions [32], through promoting inquiry, connection, and opportunities to learn and grow [33]. Policymakers can incentivize, and researchers can find ways to enhance, both of these organizational actions – protecting the delivery of EBPs, and actively engaging in quality improvement efforts – in two ways.

First, most EBPs are associated with higher marginal costs that need to be reimbursed. These costs are generated by providers, organizations, and state agencies, and several of these costs accrue to organizations. Examples include the costs of training in EBPs, costs of ongoing supervision and case consultation, productivity losses as novice clinicians gain mastery in new interventions, and the costs of documentation and regulatory compliance. Existing reimbursement strategies rarely cover these higher costs, which are currently borne largely by organizations. By developing enhanced reimbursement strategies [34] that cover the marginal costs of implementation, mental health purchasers can ensure a more sustainable organizational context for EBPs.

Second, much of organizational learning occurs through continuing education and related professional development activities by its licensed professionals. Regulations surrounding mandated continuing education units (CEUs) offer policymakers the ability to shape professional practice toward EBPs. State licensing board regulators, or their interagency partners, can assume all costs of, or subsidize, certain CEUs, provide direct technical assistance in developing courses and programs, or disallow certain courses for licensing credit. However, in order to promote an EBP environment, licensing boards will need to reconsider the structure of the CEU. Because single-shot training and didactic approaches are usually ineffective in shaping provider behavior, licensing boards will need to support quality improvement approaches that are rooted in the literature on provider behavioral change [35-38]. This literature suggests that provider education needs to be combined with auditing and feedback, as well as reminder systems and real-time decision support in order to be truly effective [38]. Re-engineering CEUs can help address an issue raised in a recent report issued by a National Institute of Mental Health (NIMH) workgroup, which noted a dearth of professionals adequately trained to provide EBPs [39].

### Policy levers at the regulatory and purchasing agency level

Regulatory and purchasing agencies form the immediate policy context for organizational activity in mental health, and have a long history of quality improvement. First-generation efforts undertaken by purchasers focused largely on profiling providers and hospitals. For example, Congress required the establishment of the National Provider Data Bank (which assists credential review of providers by state licensing boards, hospitals, and other health care entities) [40], and over 30 states make available physician profiles to the public [41]. Second-generation efforts directed at quality improvement largely involved managing quantity. For example, the rise of Medicaid managed care saw the increasing use of utilization review (or management) covering a variety of inpatient and ambulatory services [42].

Third-generation efforts, currently underway in almost all states, are designed to enhance the appropriateness of care by monitoring the type of care that is delivered, and can serve as models to support the implementation of EBPs. States can influence the type of care they pay for by using contractual requirements during the bidding process for purchase of services. In general, states tend to use five principal types of fiscal incentives – pay-for-performance or other payment incentive mechanisms, reduction in oversight and other regulatory requirements, fast-tracking or providing other advantages in the competitive bidding process, paying for infrastructure (such as free training in EBPs), and some sort of public recognition or award for providing EBPs [43]. The Ohio Departments of Mental Health and Alcohol and Drug Addiction Services have established coordinating centers to provide training, supervision, consultation, and other types of information sharing to support implementation of EBPs [44]. How a state structures its contracts to purchase EBPs is likely to be highly idiosyncratic, requiring a mix of financing and regulatory change, addressing issues of leadership and organizational politics, and ensuring training and data management efforts [45].

Second, some states have undertaken quality improvement within a disease management framework, defined as '... a system of coordinated healthcare interventions and communications for populations with conditions in which patient self-care efforts are substantial' [46]. While the specific components of a disease management program vary, common elements of all such programs include a systematic way of identifying patients; matching the intervention with their needs; ensuring the availability of EBP guidelines for not only physicians but also for all other providers involved in the care of the disease; developing an individualized treatment plan for the unique needs of the patient; designing services that promote patient adherence to this individualized treatment plan through such mechanisms as patient education, monitoring and reminders, and behavior modification programs; systematically collecting process and outcome measures; and providing a way for physicians, other providers, and the patient to obtain ongoing feedback on how care is progressing and what outcomes are being met [47]. Successful implementation of disease management programs in commercial health plans led to the establishment of the first Medicaid disease management program – Virginia's program for asthma – in the early 1990s [46]. Florida established the nation's first disease management program for a mental disorder (major depression), and the Centers for Medicare and Medicaid Services (CMS) have funded a national disease management demonstration project [48]. Disease management programs in public mental health settings are most likely to be successful for conditions that are stable over time, that can be reliably identified through screening instruments, that have well-developed and tested interventions suitable for implementation, and that require a comprehensive array of services. Conditions such as childhood trauma, for example, offer an opportunity for the construction of disease management programs, which can serve as a vehicle for the implementation of EBPs for this condition [49,50].

Third, prior authorization – the requirement that providers obtain approval for the use of a particular intervention or drug – is an existing approach used by over 30 states [51]. Originally developed as a cost-containment measure to control pharmacy costs, prior authorization is often combined with formularies to restrict the variety of medications available to beneficiaries. Although little experience exists with prior authorization for behavioral interventions, such programs could be used to restrict potentially harmful interventions being delivered to enrollees. Conversely, eliminating evidence-based interventions from restrictions on session limits, modifying designated patient regulations that restrict who can receive the service, and modifying existing regulations governing session lengths can all serve to promote EBPs using this approach.

Fourth, many states have experience with provider profiling, which can be used to promote an EBP agenda. Attempts to improve the appropriateness of psychotropic medication use, for example, have seen the use of provider-level audits of prescriptions, a practice called prescriber profiling. While attempts are underway to reduce sharing of prescriber data with pharmaceutical companies [52], state purchasers can, and do, access prescriber-level records to identify individuals engaging in aggressive pharmacotherapy, and to monitor compliance with established medication algorithms [53]. However, few states seem to have implemented 'psychotherapy profiling' systems, which may allow therapists with specialized training to be identified and reimbursed at a higher rate. Psychotherapy profiling will require states to develop specialized billing codes that would permit comprehensive assessments [54,55], which could guide deployment of EBPs. States may also need to modify their billing requirements to accommodate EBPs that differ structurally from individual or group therapy.

Finally, states will need to find a way to link all such procedural efforts with individual client-level outcomes. Outcome measures in mental health are difficult and expensive to administer, which is why most quality improvement efforts focus on performance or process indices. However, federal policymaking seems to be moving toward requiring comprehensive outcome measures as a condition of payment – such as the requirement that all home health agencies seeking Medicare certification report on a set of common measures contained in the Outcome and Assessment Information Set (OASIS) [56]. SAMHSA's national outcome measures (NOMs) also outline a series of client-level as well as systemic outcomes across ten domains [29], although there is not yet a link between purchasing and attainment of these measures. To date, few states have established mechanisms to collect data on performance indicators statewide [29], and none have established statewide client-level outcomes monitoring. Developing or adopting outcomes assessment, either statewide or within a given system (*e.g*. children's mental health) is a necessary first step for any outcomes-based reimbursement approach as a tool to support EBP implementation.

### Policy levers at the political level

We define the political context of EBP implementation as involving all legislative and advocacy efforts that support such a goal. While few laws are directed specifically at implementation efforts, legislation often forms the enabling resource for EBP implementation. For example, following Oregon's 2003 legislation (discussed earlier), the Iowa legislature passed in 2004 a law that extended an EBP mandate beyond state agencies, requiring community mental health centers to spend an increasing share of Mental Health Block Grant dollars in purchasing EBPs [57]. Policymakers will need to carefully weigh the nature of the evidence, the availability of local resources to deliver the EBP with fidelity, and the unintended consequences of micromanaging care before considering such legislative strategies.

While much of the focus of EBP activists has been on such targeted laws, other laws, such as mental health parity laws, also require attention by state policymakers. These laws have a broader objective – ensuring that mental health services can be adequately resourced and delivered – which is a necessary requisite for providing EBPs. The issue of mental health parity extends far beyond providing necessary resources, however, in being intimately related to social-level issues of stigma, misunderstanding, and discrimination toward people with mental health diagnoses [58]. Parity legislation, therefore, is necessary but insufficient to drive implementation efforts due to these societal factors, which we consider in greater detail below.

Third, another way in which state legislatures can incentivize EBP implementation is by passing laws that reduce or forgive educational debts for professionals who adopt and promote EBPs. Debt forgiveness is currently used to promote health sciences, counseling, and social work practice in high-need areas with particularly vulnerable populations, based on federal legislation [59-61]. By re-conceptualizing an underserved area as extending beyond geography to types of practice, legislation could provide forgiveness of educational debt to clinicians delivering EBPs to defined populations, and make EBP-trained clinicians more financially attractive to employers.

Fourth, policymakers can begin to reduce or eliminate structural stigma. Structural stigma refers to state policies or legislation that deliberately deprive certain groups of individuals (in this case, the mentally ill) from the rights and privileges that accrue to other groups. A 1999 survey of state laws revealed that 44 states imposed some restrictions on the rights of mentally ill individuals to serve on a jury, 37 imposed restrictions on their voting, 23 imposed restrictions on their holding elective office, and 27 imposed restrictions on their parental rights [62]. An analysis of the 968 mental health bills introduced into legislatures nationwide in calendar year 2002 revealed that although most states were legislating to protect the rights of mentally ill individuals, some states were expanding restrictions of parental rights for the mentally ill [63]. While we are unaware of examples of structural stigma that affect EBPs, legislators will need to pay close attention to the effects of their EBP-related lawmaking in order to avoid discriminating against mental health consumers.

Fifth, policymakers who look to support for an EBP agenda from consumer-focused advocacy groups will find that these groups have not been universally supportive of the emergence of EBPs. On one hand, there is an appreciation of a model of practice built upon documented results rather than on the opinions of experts [64], and organizations such as the National Alliance for the Mentally Ill have supported some EBP implementation efforts [65]. Professional organizations are generally strong supporters of EBPs, have testified before Congress [66], and have set up task forces to promote such practices [67], among other efforts. Conversely, consumer-directed advocacy groups, such as the National Mental Health Consumer's Association, have advanced equivocal positions [65]. Several family/consumer fears have been documented in the literature [7], and include concerns that the EBP movement is insufficiently aligned with the recovery model (in which mental health consumers are presumed capable of making considerable progress toward independence [68]), that EBPs may replace other needed services, that there may be a lack of availability of providers able to deliver EBPs, that EBPs may be unduly prescriptive and cause consumers to lose control over their care, and that they may not be sufficiently culturally competent [6,69].

Policymakers who confront such issues may benefit from an approach to engaging consumers and families proposed by Birkel and colleagues [70], who suggest that EBP implementation efforts should actively build collaborative relationships at the beginning of the implementation process; should find ways to integrate recovery in the development and deployment of EBPs; should pay special attention to racial/ethnic, geographical, cultural and linguistic diversity in all implementation efforts; and should develop and disseminate resources to support not just the EBP but also its advocacy. Such an inclusive approach to policymaking that takes into account diverse consumer and family needs may be necessary to assure widespread acceptability of EBPs.

### Policy levers at the social level

Several efforts discussed above, including those focused on consumer advocacy, cultural competence, and consensus-building, lie at the interface between political and social contexts of EBP implementation. In this section, we focus on how policymakers can mitigate the effects of stigma in preventing access to EBPs.

Combating mental health-related stigma is a goal of the President's New Freedom Commission [71], and is essential because access to EBPs is conditional upon access to mental health services. Several EBPs also require greater amounts of adherence to protocols, which can be compromised in the presence of stigma. However, empirical guidance for policymakers on ways to reduce stigma is still emerging. The NIMH established a Stigma Working Group in 1999, and has issued a program announcement to fund research projects on stigma reduction [72]. SAMHSA established its Eliminating Barriers Initiative in 2003, and funds a Resource Center to Address Discrimination and Stigma [73]. These efforts aim to identify effective approaches to reduce stigma and discrimination, such as those involving public educational programming, producing educational materials, contact-based approaches, and public service announcements. Other efforts at ending stigma use social marketing principles. For example, the World Health Organization's 2001 World Health Day launched an international campaign to reduce stigma associated with mental illnesses among youth, which included organization of school contests addressing stigma, development of teacher guides, production of a book on stigma through children's eyes, and a curriculum for use in health education programs worldwide [74,75].

While reducing stigma may be an educational endeavor, eliminating discrimination is usually a legal process. Policy approaches that can serve more proximal goals of ending discrimination include ensuring appropriate treatment and care for individuals with mental illnesses, supporting effective public and professional education, and preserving mental health allotments in health, welfare, and research budgets [76]. While none of these approaches directly serve an EBP implementation agenda, they create the contexts for improved access to mental health services, within which an EBP agenda can be supported.

## Summary

Efforts to improve the quality of mental health services should consider the larger ecology that is known to affect implementation instead of solely focusing on specific interventions and the specific locations of their delivery. An integrated approach to policymaking at several levels of this ecology – as summarized in Table 1 – can support a more sustainable, and ultimately more successful, implementation process. In addition, implementation researchers need to be aware of influences at multiple levels of this ecology; absent a conducive environment, gains from even the best-designed approaches targeted solely at individual providers or organizations are unlikely to persist over the long term. Implementation researchers will also need to build in the systematic collection of data at multiple levels of the implementation ecology while designing their studies in order to identify and test change strategies that are likely to succeed. Practice leaders, such as executive directors of mental health organizations, are usually highly attuned to the environment within which their organizations operate, and will be important sources of information on, and change agents in, this ecological approach to implementation. We recommend that all individuals involved with implementation efforts consider these strategies as they collectively strive to increase the availability of EBPs to vulnerable populations.

## Competing interests

The authors declare that they have no competing interests.

## Authors' contributions

RR conceived this manuscript, and led the writing. CLB assisted with the literature review, and wrote parts of this manuscript. ALS participated in the conceptualization and writing of this manuscript.
